# The Intelligence of Rickety Children

**Published:** 1912-04-13

**Authors:** 


### MATERNITY AND MOTHERING.

(Inquiries and Correspondence are invited.)

### The Intelligence of Rickety Children.

There is probably no disorder which affects so large a proportion of the child population as rickets. Although rickets has been dignified abroad with the title of the English disease, it is so ubiquitous and so common that it is certainly doubtful whether it is any more prevalent in Britain than on the Continent, or indeed in any other part of the civilised world. Amongst savages, too, it occurs, though less frequently than in European races; the difference is doubtless due to the universal prevalence of breast feeding in uncivilised societies.

The subject of rickets is a very large one; its causation, signs, and treatment have been made the materials for weighty treatises by eminent physicians. Into these manifold aspects of rickets it is not proposed to inquire at the moment. Our immediate purpose at present is merely to direct attention to one special association—that of the mental condition of the rachitic, which has been carefully inquired into recently by Dr. Gilmour.* This aspect of rickets has received hitherto but little attention, but it is one whose importance can easily be recognised when the great prevalence of the condition is fully appreciated.

### Occurrence in all Classes.

One is accustomed to think of rickets as a disease of the poor, as a disease due to a combination of overcrowding, insanitary dwellings, insufficient or improper food, lack of fresh air and sunshine, and, in fact, as a product of unhygienic surroundings. It is quite true that all these factors make for rickets in little children, and that severe cases of rickety deformities are met with almost entirely among the most ignorant and poorest sections of the community. This, however, may be, and very likely is, due to the fact that in the upper and middle classes treatment is undertaken much earlier; in other words, that neglected cases are rare. Certainly the percentage of children, even among the wealthy, in whom evidences of slight rickets can be detected, is much larger than laymen have any conception of. Usually the practitioner orders appropriate treatment without confiding to the parents his inward opinion of the exact lesion he has diagnosed. Some consternation was aroused a few years ago when the well-known medical officer of a celebrated public school stated that 50 per cent. of the boys showed evidences of rickets in infancy. This estimate is larger than most medical men would be inclined to give; but at any rate, it shows how extremely common mild rickets is, even among the educated and well-to-do classes.

In the Scottish elementary schools, whence Dr. Gilmour has drawn his figures, the percentages were 23 for boys and 12 for girls in a series of over 6,000 cases. In Scotland the diet of the labouring classes has always been more wholesome, if also more frugal, than among the corresponding class in England; and breast feeding has not been abandoned to the same extent as in this country.

### Mental Defects in the Rickety.

Many interesting statistics are given as a result of the extremely searching inquiries made into family history, home environment, and physical characteristics of the affected and non-affected children. For the moment we may turn to the deductions founded on the figures concerning the mental condition of the rickety as compared with that of the non-rickety child. The subject is, as the author says, complex. Sometimes there are factors which may underlie both mental enfeeblement and physical illness (such as rickets); and parents will be especially desirous of concealing shortcomings of this kind. Allowing for this, it still appears a safe conclusion that there is a higher percentage of the mentally dull among the rickety than among the others. Such dulness seems to exist in children in whom no hereditary taint or evidence of neglect and malnutrition can be found.

The incidence of the symptoms and signs of rickets varies considerably: sometimes the legs may show advanced deformity when the head, ribs, and epiphyses of the arms show but slight changes; sometimes it is the other way about. It is therefore reasonable to suppose that if rickets can at times affect the nutrition of the brain, it may occasionally affect it severely, even though generally the result is imperceptible. It would also follow that the amount of bony change is not necessarily any guide to the amount of disturbance of the cerebrum; and this conclusion is entirely confirmed by Dr. Gilmour's figures obtained from his inspections of the children themselves. It is somewhat consoling that he believes the impairment of mental power is not usually permanent, but improves as the physical condition of the child improves; though the subsequent rate of progress is slightly slower than that of the healthy child.

### Mental Defect may become Permanent.

On the other hand, when the family history, in respect of alcoholism or degeneracy of any kind, is a bad one, the impairment is liable to become permanent, even when there is improvement in the physical condition of the child. In fact, it is probable that in tainted families rickets may be the deciding factor in completing the wreckage of a mind which in a body of unimpaired health might have triumphed over its handicap and remained sound.

The problems of rickets are many and pressing. The disease is one which is entirely preventable; it is a disease solely of ignorance, and as such it is a blot upon our civilisation. Hitherto its purely physical results have received most consideration; they are serious enough, in all conscience, but it may be doubted whether, after all, the mental effects upon the young children of a large section of the nation may not be more important still.

* *School Hygiene*, February 1912, p. 6.

